# Assembly of Carbon Dots into Frameworks with Enhanced Stability and Antibacterial Activity

**DOI:** 10.1186/s11671-021-03582-3

**Published:** 2021-07-29

**Authors:** Pengfei Zhuang, Kuo Li, Daoyong Li, Haixia Qiao, Yifeng E, Mingqun Wang, Jiachen Sun, Xifan Mei, Dan Li

**Affiliations:** 1 Jinzhou Medical University, Jinzhou, China; 2 Department of Basic Science, Jinzhou Medical University, Jinzhou, China; 3 Department of Pharmacology, Jinzhou Medical University, Jinzhou, China; 4 Department of Basic Medical Science, Jinzhou Medical University, Jinzhou, China

**Keywords:** Carbon dots, Carbon dots-based frameworks, Bacteria, *E. coli*, *S. aureus*

## Abstract

Carbon dots (CDs) have been widely used as antimicrobials due to their active surface, but some CDs suffer instability. Therefore, the relative applications such as the antibacterial activity may not be reliable for long-term use. Herein, we synthesize CDs with blue fluorescence by a hydrothermal process. Thereafter, polyethylenimine was applied for the assembly of CDs into CDs-based frameworks (CDFs). The CDFs exhibited quenched fluorescence but showed more stable properties based on the scanning electron microscope and zeta potential investigations. Both CDs and CDFs show antibacterial activity toward Gram-negative Escherichia coli (*E. coli*) and Gram-positive *Staphylococcus aureus* (*S. aureus*), but CDFs exhibited better antibacterial performance, and *S. aureus* could be completely inhibited with the minimum inhibitory concentration of 30 μg/mL. This reveals CDFs magnify both the stability and antibacterial activity, which would be more promising for practical applications.

**Graphic abstract:**

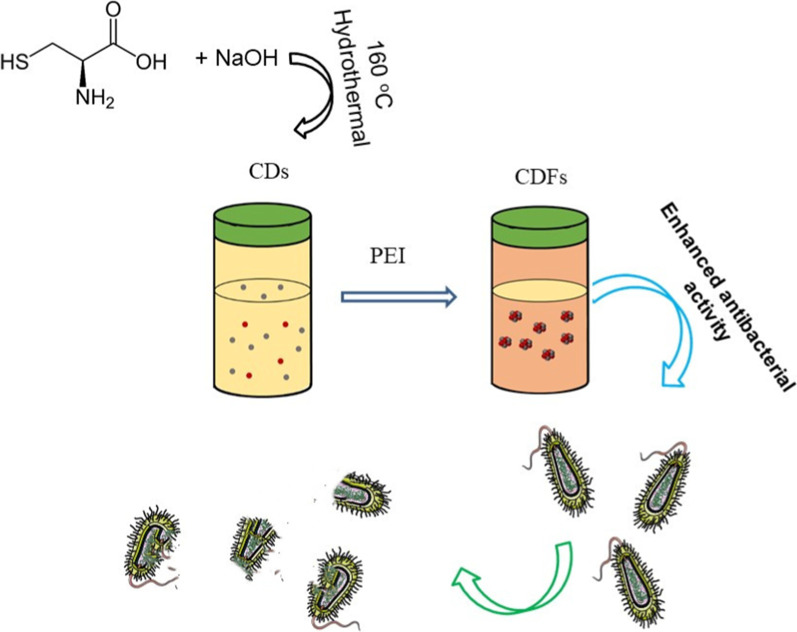

**Supplementary Information:**

The online version contains supplementary material available at 10.1186/s11671-021-03582-3.

## Introduction

Bacterial infections show a serious threat to human lives, and the development of effective medicines to disinfect bacteria is in great demand [1]. Various antibiotics have been used for treating bacterial infections, but the overuse of antibiotics causes other problems such as side effects and drug-resistant issues [2]. The nanomaterials including antimicrobial polymers [3], metal nanomaterials [4], and carbon nanomaterials [5, 6] have been used as alternatives to classical antibiotics [7]. Both drug-resistant and toxic problems are relieving [8]. Recently, CDs [9, 10] and nanoclusters (NCs) [11] are well applied for combating bacterial infections because they are biocompatible [12], active [13], and can be easily cleaned by circulations due to the ultra-small sizes [14, 15]. Especially, researchers have found that CDs show excellent free radical scavenging ability, which can be stronger than many traditional anti-infection drugs [16–18]. However, some ultra-small antimicrobials suffer poor stability due to the larger oxidative surface area [19]. It is highly desired to develop more effective antibacterial agents for combating bacterial infections for long-term use.

**Fig. 1:**
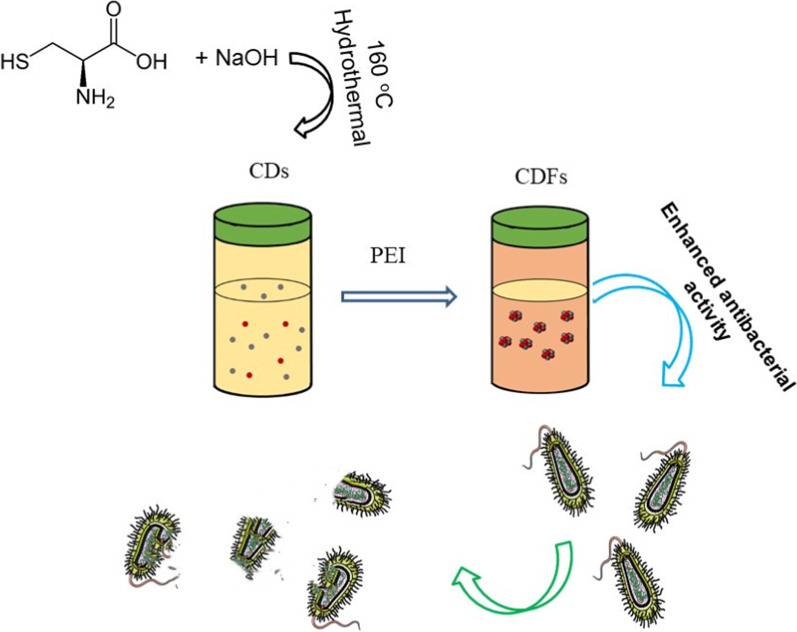
Scheme for the synthesis of CDs and the assembly into CDFs by adding PEI with enhanced antibacterial activity

To meet the demand for practical applications, the antimicrobials should have the following characteristics: (a) Excellent stability remains unchanged for a certain time in an ambient environment; (b) Excellent biocompatibility and low toxicity: (c) high antibacterial activity. The larger nanomaterials tend to be more stable, but they might have relatively weaker antibacterial activity due to the smaller active surface area. Considering both the weakness of small and large nanomaterials, we report the assembly of the small CDs into large CDFs by simply adding polyethyleneimine (PEI) (Fig. 1). CDs were not fused but kept their morphology as building blocks. Therefore, the entire CDFs showed larger sizes but demonstrated more excellent stability without losing the active properties of CDs. Further, we found the CDFs displayed enhanced antibacterial activities against both Gram-negative Escherichia coli (*E. coli*) and Gram-positive *Staphylococcus aureus* (*S. aureus*) compared to CDs, indicating their broad-spectrum antibacterial performance, though many CDs only eradicated Gram-positive bacteria [20]. In addition, the CDFs promoted the proliferation of the PC12 cells (a cell line obtained from a pheochromocytoma of the rat adrenal medulla), showing great potential for nerve recovery applications [21]. This work suggests the assembly of small CDs into large CDFs not only enhances the stability but also magnifies the antibacterial activity.

## Materials and Methods

### Materials and Instrument

X-ray surface photoelectron spectra (XPS) were recorded on an ESCALAB250Xi X-ray surface photoelectron spectroscopy (XPS) instrument. The transmission electron microscope (TEM) was performed by a JEM-2100 microscope operating at 200 kV. The fluorescence of the materials was obtained using the F97 fluorescence spectrometer. The FDA/PI staining of the bacterial cells was recorded in tapping mode with a Leica DFC450C microscope. Fluorescence lifetime was measured on a time-correlated single-photon counting (TCSPC) system using a Nanolog spectrofluorometer (Horbia JY, Japan). Ultraviolet–visible spectroscopy (UV–vis) spectra are obtained from the UV-1600 instrument. The bacterial imaging was observed by a confocal microscope (Olympus FLUOVIEW FV1000 c). All the reagents were of analytical grades. The deionized water was used through the experiments. Cell counting kit-8 was obtained from Beyotime Biotechnology.

### Preparation of CDs and CDFs

L- cysteine (1.0 g) was dissolved in 10.0 mL of deionized water and mixed well. Then, the pH of the solution was adjusted to 9.0 with 1.0 M NaOH. The solution was transferred to a hydrothermal reactor and heated at 160 °C for 24 h. After the solution was cooled to room temperature, the resulting solution was subjected to dialysis using a dialysis bag (MW 7000 cut off) for one day. The as-obtained CDs were used for the following characterizations and experiments. For the preparation of CDFs, 40 µL of 1% PEI was added to 1 mL of the CDs. The mixture was allowed to stay for 1 h. The product was purified by dialysis using the same method as the purification of CDs. For HR-TEM characterization, the samples were concentrated into a small volume, transferred to a silica gel column, and eluted with methanol and dichloromethane to obtain the further purified products.

### Toxicity Evaluation

PC12 cells were seeded in 96-well plates for 12 h and then were incubated with CDs and CDFs with different concentrations. The number of viable cells was investigated using a Cell Counting Kit-8 assay (CCK-8). 3-(4, 5-Dimethylthiazol-2-yl)-2, 5-diphenyltetrazolium bromide (MTT) (5 mg/mL in PBS) was added at 1/10 culture volume, and the cells were returned to the incubator. After that the supernatants were discarded and 200 μL of dimethyl sulfoxide (DMSO) was added to each well. The crystals were dissolved by shaking the plates for 10 min. The absorbance at 490 nm was measured using the Microreader (Varioskan LUX Multimode Reader). Blank control wells were included for all the absorbance measurements.

### Antibacterial Experiment

*E. coli* and *S. aureus* were incubated in the absence and presence of CDs and CDFs at 37 °C with 250 rpm shake. Growth of the bacterial cells in Lysogeny broth (LB) culture was measured by the Microreader at 600 nm wavelength (OD600). LB medium was used as the blank control. The OD600 stands for the cell density, and the relative cell viability was calculated based on the comparison between the cultured bacterial cells in the presence of the materials to the control group (OD600 of the bacterial cells in the absence of the CDs or CDFs). The live/dead bacteria are evaluated by the FDA/PI staining protocol [22].

### Detection of Ros Reactive Oxygen Species (ROS)

Intracellular ROS production was measured in bacterial cells before and after the treatment by CDs and CDFs for 12 h, with a 2′, 7′-dichlorofluorescin diacetate (DCFH-DA) probe following the instructions. The fluorescence was detected at an emission wavelength of 525 nm with excitation at 488 nm.

## Results and Discussions

### Characterization of the Materials

#### Size Investigations

**Fig. 2:**
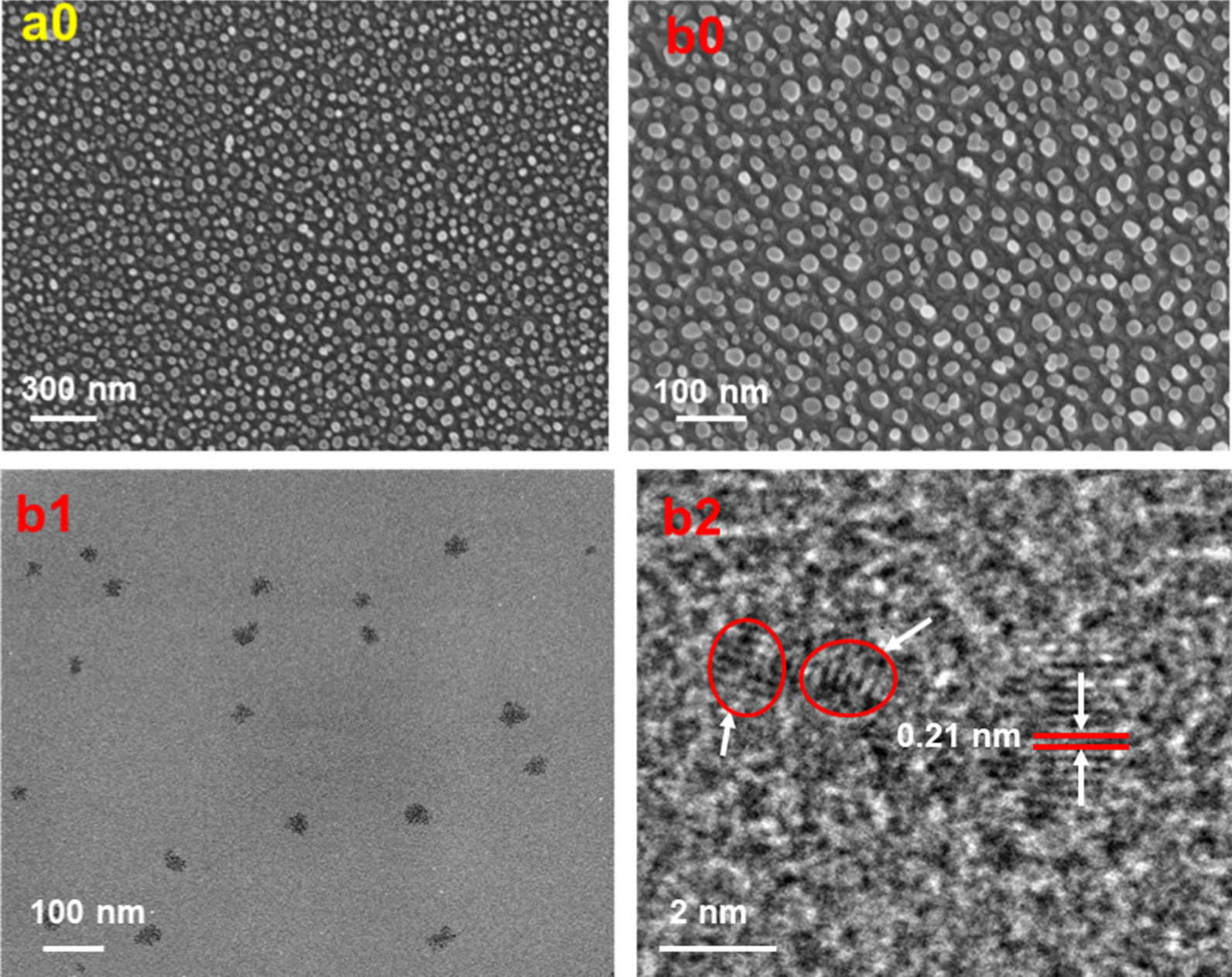
SEM for CDFs with relatively lower (**a0**) and higher magnification (**b0**); TEM (**b1**) HR-TEM (**b2**) of CDFs

Figure 2 shows the SEM of the powder for CDFs with relatively lower (Fig.  2a0) and higher magnification (Fig. 2b0) after exposure to the air for several hours. It can be seen that the CDFs were well distributed and showed an average size of ca. 25 nm. CDs were supposed to show mono-dispersed small sizes based on the TEM and AFM study (Additional file 1: Figure S1), but these small particles exhibited aggregation observing by SEM after exposure to air for a certain time (Additional file 1: Figure S2). On the other hand, CDFs kept their morphology though they were exposed to the ambient environment. This indicated the as-obtained CDFs are more promising for practical applications. CDFs were further characterized by TEM (Fig. 2b1). It can be seen that CDFs exhibited assembly structures with small CDs as the building blocks. The high-resolution transmission electron microscopy (HR-TEM) (Fig. 2b2) analysis revealed that the building block of CDFs exhibited lattice fringes with a d-spacing of 0.21 nm, corresponding to the (100) plane lattice of graphite, which was similar to the classical CDs [23–25]. Therefore, the assembly CDFs do not lose the feature of CDs, which may combine the advantages of both the entire large frameworks and the interior small building blocks.

#### Zeta Potential

**Fig. 3:**
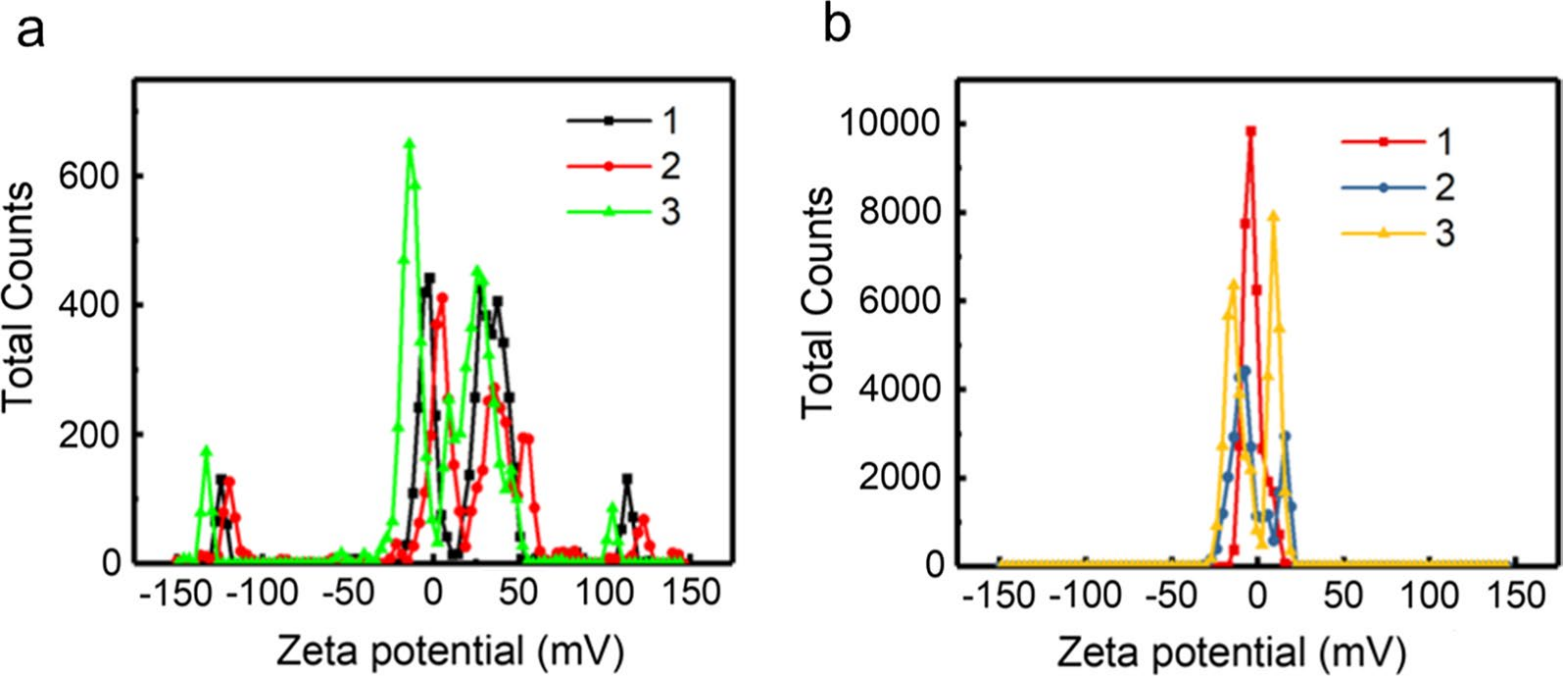
Zeta potential of CDs (**a**) and CDFs (**b**)

Zeta-potential investigations were used to measure the degree of electrostatic repulsion of CDs and CDFs between adjacent charged particles in the dispersed system (Fig. 3). It can be seen that the zeta-potential peak of CDs was not well featured, which were exposed to the ambient environment. Besides, multiple zeta potential peaks were obtained with large zeta deviations (Table 1), indicating the CDs were quite unstable and unrepeatable. On the other hand, the zeta potential peak for CDFs focused at a relatively stable range. We also had much smaller zeta deviations based on three times measurements, revealing CDFs were more stable and the dispersed systems had samples with higher purity.

**Table 1:** Zeta potential (ZP) and Zeta deviation (ZD) of CDs and CDFs

Samples	ZP1 (mV)	ZD 1 (mV)	ZP 2 (mV)	ZD2 (mV)	ZPP 3 (mV)	ZD3
CDs	4.10	70.5	4.39	76.2	2.13	45.7
CDFs	− 2.65	5.63	− 3.23	12.1	− 2.91	12.5

#### Fluorescence Properties

**Fig. 4:**
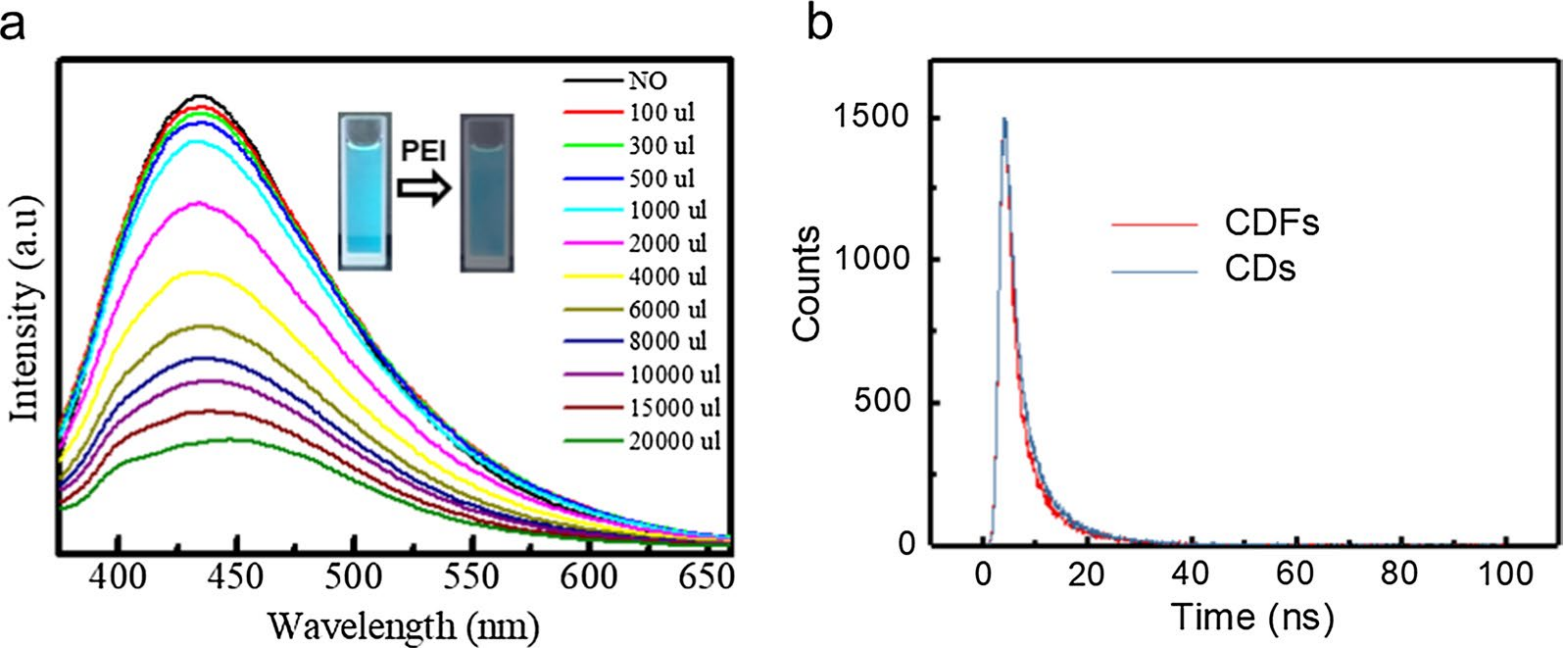
Fluorescence emission spectra **a** of CDs as the titration of PEI, and lifetime **b** for CDs and CDFs

The fluorescence emission spectra were used to monitor the assembly process of CDs to CDFs (Fig. 4). As the titration of PEI, the fluorescence emission at 350 nm gradually quenched (Fig. 4a). But no significant fluorescence spectra shift was observed, revealing no aggregation occurred. The assembly structure of CDFs changes the entire size of CDs, which influences fluorescence. Meanwhile, the nearby CDs marked the fluorescence of each other. As well as this, surface chemistry plays an important role in fluorescence properties. The nitrogen atoms and sulfur atoms on the surface of CDs can generate energy traps. The bright fluorescence of CDs attributes to the defect surface with many carbonyl and amino groups. After the functionalization of PEI, the surface of CDFs was dominated by the amino groups, which quenched the fluorescence together with the growing sizes. The comparison of the fluorescence behavior of CDs and CDFs was further examined by TCSPC to understand the photo-generated charge recombination pathways of the materials (Fig. 4b). Emission was monitored at 430 nm. The fluorescence decays required a two-component exponential fit. The time constants and relative amplitudes are fitted and summarized in Table S1. It could be seen that the lifetime of the dominant component for CDs was 2.45 ns, while that of the other component was 7.47 ns. On the other hand, the lifetime of the dominant component for CDs was 1.98 ns, while the other component showed a lifetime of 7.30 ns. No significant lifetime change was observed between CDs and CDFs, which also indicated the properties of CDs was not substantially changed while assembly into CDFs [26].

### Toxicity

**Fig. 5:**
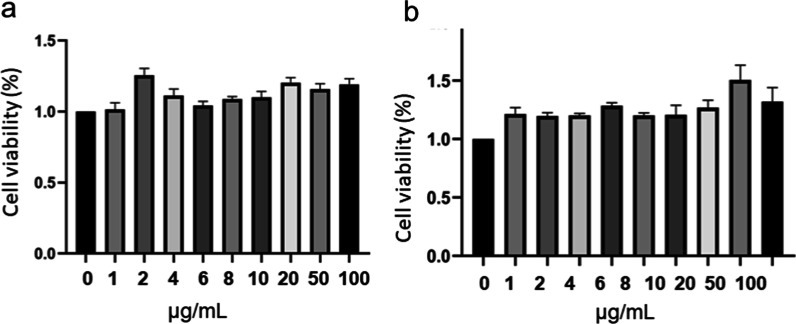
Cell viability of PC12 in the presence of CDs (**a**) and CDFs (**b**). Cell viability (%) = (absorbance of the experimental group—absorbance of the blank group)/(absorbance of the control group—absorbance of the blank group) × 100%

For evaluation of the safety of the materials, the toxicity of the CDs and CDFs to PC12 cells is investigated. Assays of MTT were conducted to investigate the influence of the materials on cell viabilities (Fig. 5). After incubation of PC12 with CDs and CDFs, cell viabilities were not much affected within 24 h. Interestingly, both carbon materials promote the proliferation of PC12, which plays an important role in the therapy of nerve damage. These results indicate the low toxicity of the materials, and the materials are promising for nerve protection with PC12 cells involved [27].

### Antibacterial Investigations

**Fig. 6:**
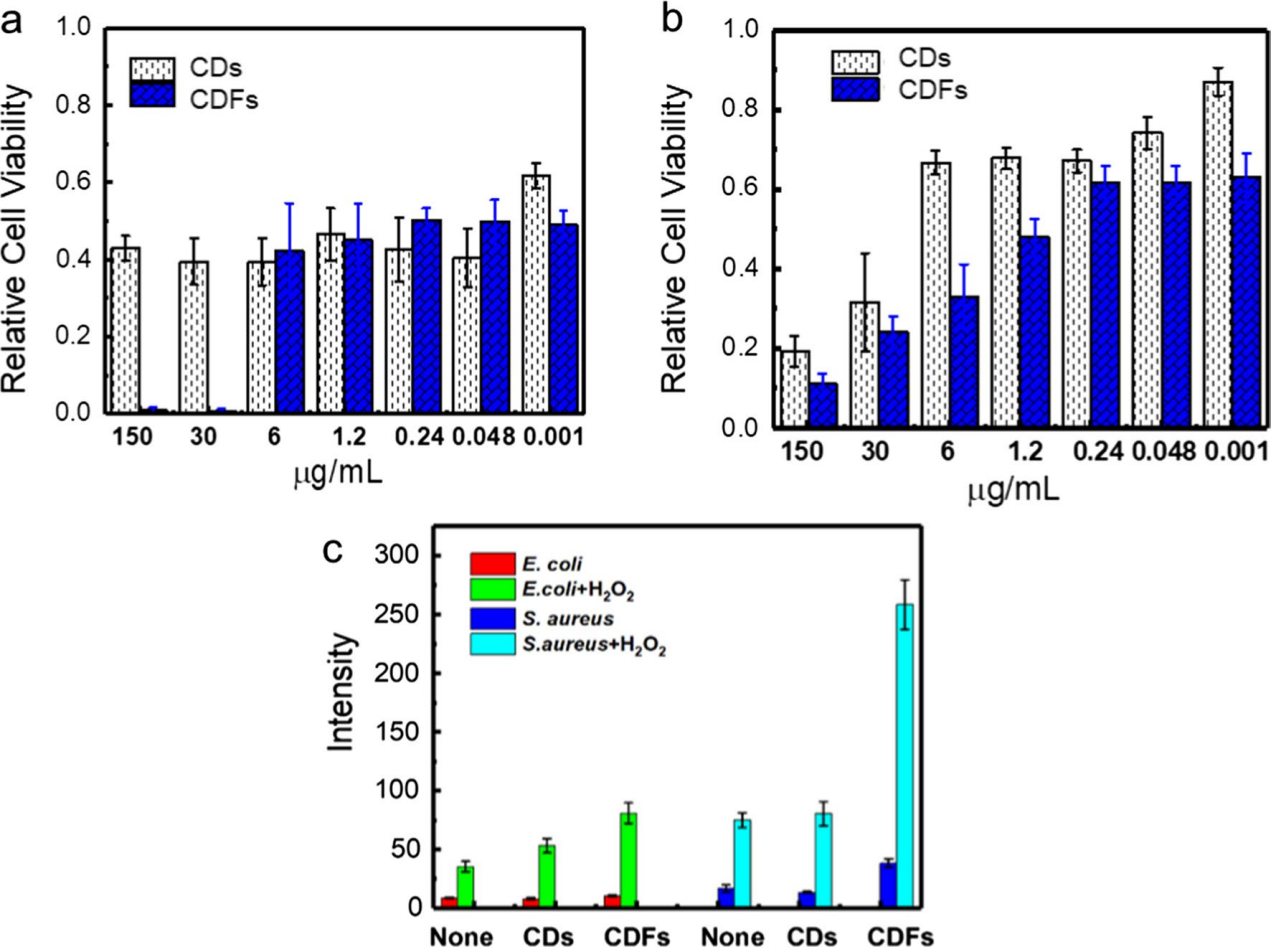
Antibacterial activity of CDs and CDFs against *S. aureus* (**a**) and *E.coli* (**b**), **c** fluorescence intensity of DCF at 525 nm, which shows a linear relationship with the ROS level, with the treatment of CDs and CDFs in the absence and presence of H_2_O_2_ (100 μM)

The antibacterial activities of the CDs and CDFs were initially evaluated by measuring the bacterial density in the presence of these agents at 600 nm [28]. Fig. 6 shows a substantial antibacterial effect of both the CDs and CDFs against both the *S. aureus* and *E. coli* cells. Especially, the viability of the *S. aureus* cells was almost 0 when larger than 30 µg/mL of the CDFs were used. Similarly, the *E. coli* cells were disinfected by both CDs and CDFs. CDs showed multiple charges (Fig. 3a). On the other hand, the CDFs were with insignificant charges (Fig. 3b), which might suppress the bacterial adhesion under weak repulsion thus interacting with the bacterial surface more easily. By comparison, the CDFs show higher antibacterial activity based on the phenomenon that a relatively larger ratio of the cells is killed when larger than 6 µg/mL of the materials are used. The enhanced antibacterial activity of CDFs is possibly attributed to the synergy effect of CDs as building blocks as well as the more stable surface charges. To deeply understand the antibacterial mechanism, the ROS that could oxidize nonfluorescent DCFH to fluorescent DFC was measured (Fig. 6C). Both carbon materials did not significantly induce ROS production after treating *E. coli*. However, CDFs remarkably enhanced intracellular ROS while interacted with *S. aureus*. Since ROS can damage bacterial DNA, RNA, and proteins, the enhanced value facilitated the disinfection of the bacteria. Furthermore, the ROS was stimulated with H_2_O_2._ It is worth noting that ROS productions were all dramatically promoted compared to the H_2_O_2_ treatment alone, while CDFs showed the highest enhancement. This indicated the combination of H_2_O_2_ with these two carbon materials might further enhance the antibacterial activity, especially for the CDFs.

Some CDs were previously reported for disinfecting *S. aureus*, which is listed in Table 2 for comparison. The current CDFs showed a competitive MIC. Besides, rather than only decreasing the investigated cell viability, CDFs promoted cell proliferation of PC12, indicating their versatility while treating bacterial infections.

**Table 2:** Synthesis of CDs and the eradication of S. aureus

Name	Synthesis condition	MIC	Cytotoxicity	Refs.
CQDs	Hydrothermal method using ammonium citrate at 180 ℃	50 μg/mL	Several cancer cells showed little cytotoxicity response (cell viability > 90%) with 50 μg/mL CQDs	[29]
CDs	Hydrothermal method using m-aminophenol and tartaric acid at 180 ℃	250 μg/mL	The cell viability of HeLa cells exceeds 70% at 400 μg/mL of CDs	[16]
ACDs	The smoke of A. argyi leaves from burning was collected and filtered	*S. aureus* was not completely inhibited by ACDs of 150 μg/mL	85% of 293 T cells survived at 150 μg/mL of ACDs	
CDFs	Hydrothermal method using L-cysteine and NaOH at 160 ℃	30 μg/mL	CDFs promoted PC12 cell proliferation. Thus, the cell viability is larger than 100%, indicating the nerve protection potential	Current work

CQDs, carbon quantum dots; 293 T cells, the human embryonic kidney (HEK) 293 T human cells

**Fig. 7:**
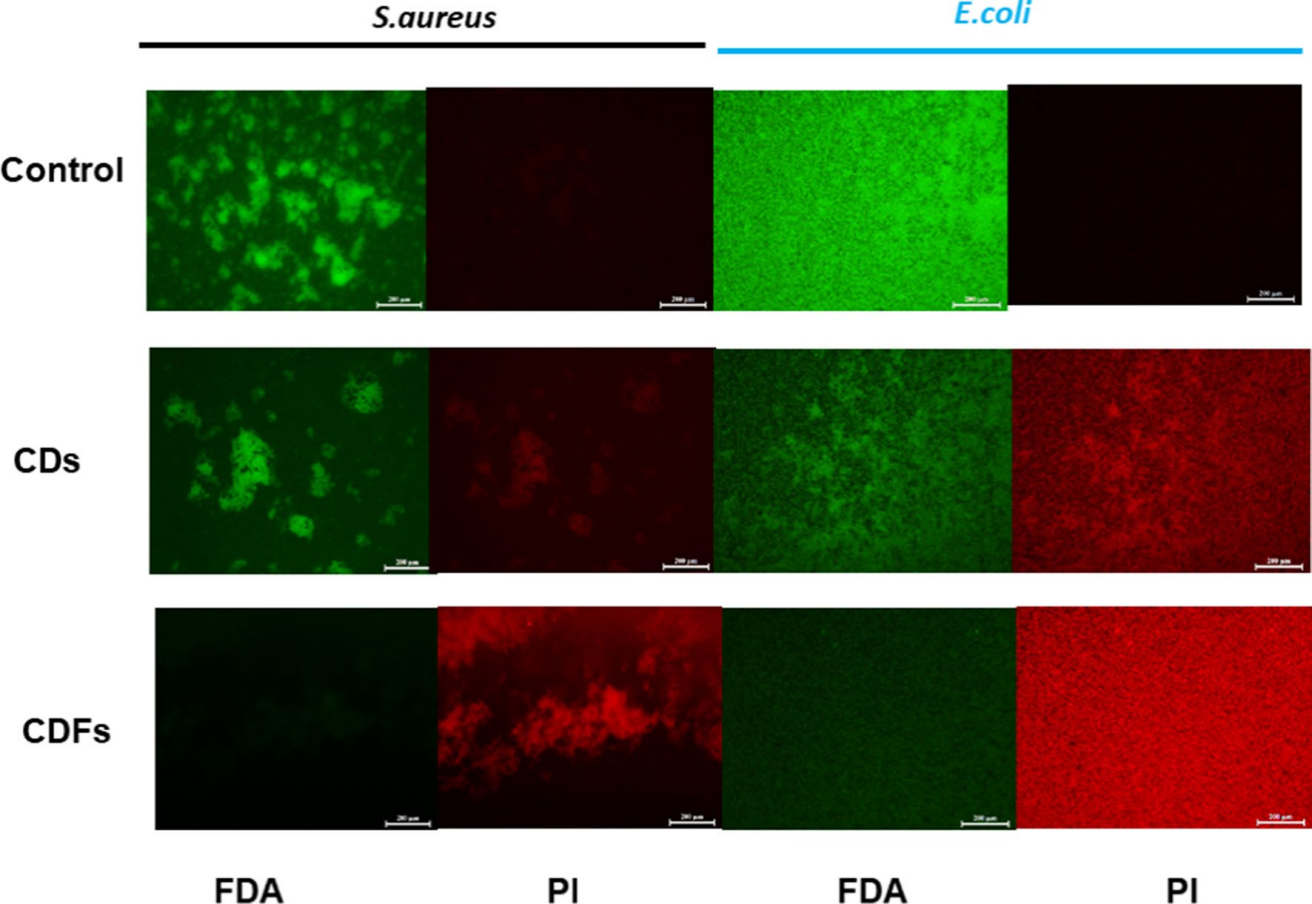
FDA/PI staining *S. aureus* and *E. coli* in the absence and presence of CDs and CDFs at 30 µg/mL. Scale bar, 200 µm

The integrity of the bacterial membrane after the CDs and CDFs treatment was investigated by the live/dead staining experiment (Fig. 7). The green fluorescent FDA staining can only show the live cells, while red fluorescent PI specifically stains dead bacteria with broken membranes, but the live ones with intact bacterial membranes are unstained [30, 31]. As shown in the fluorescence images, obvious red fluorescence was seen in the CDs-treated samples and a much higher density of the dead cells was seen by the CDFs-treated samples.

**Fig. 8:**
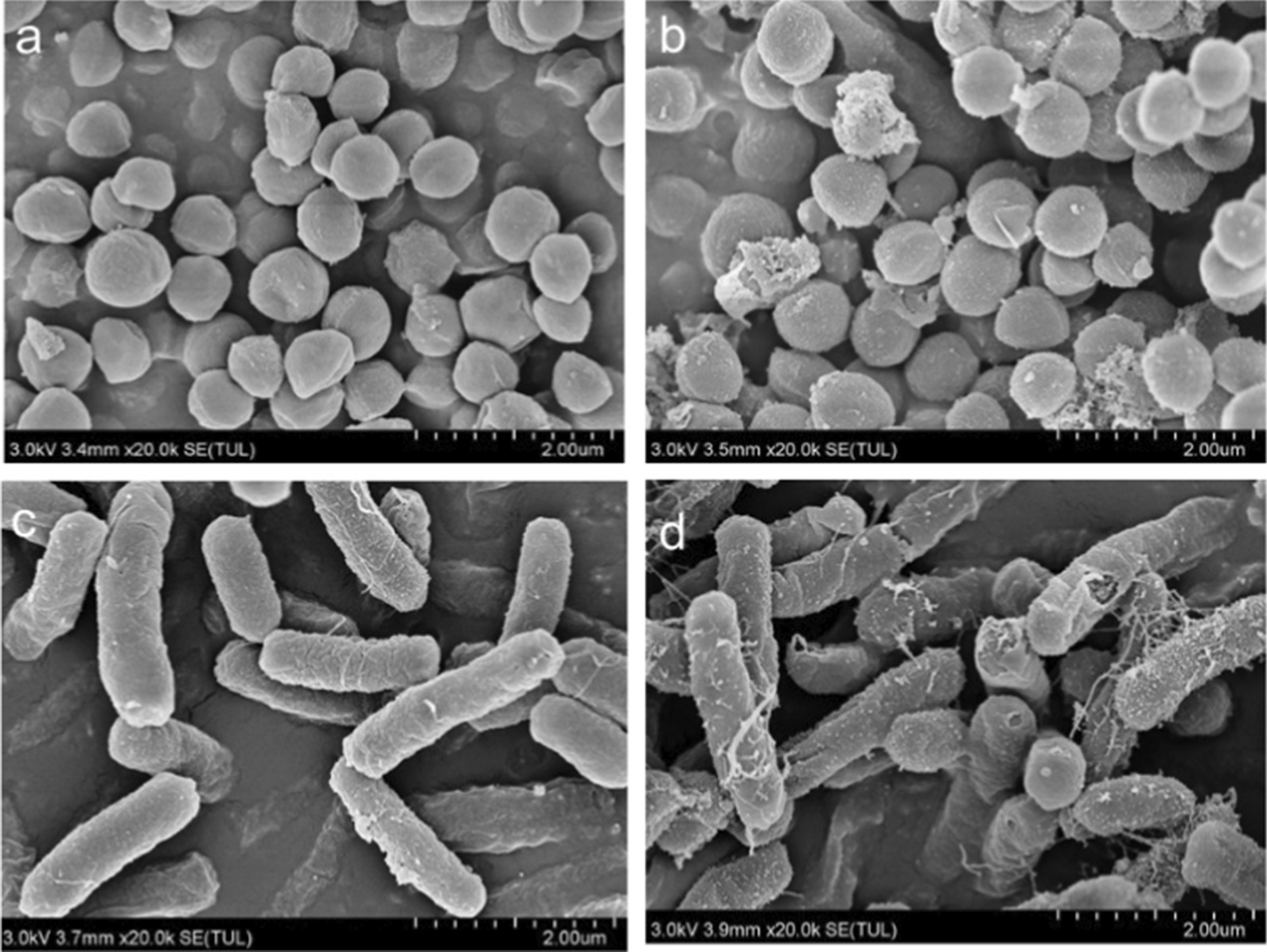
SEM for *S. aureus* (**a**, **b**) and *E.coli* before (**a**, **c**) and after (**b**, **d**) the treatment with 30 µg/mL of CDFs. Scale bar: 2 µm

Based on the above comparisons, we conclude that CDFs are promising for killing both Gram-positive and Gram-negative bacterial cells. Therefore, *E. coli* and *S. aureus* before and after treating with 30 µg/mL of CDFs were characterized by SEM (Fig. 8). As shown in Fig. 8a, c, bacteria before treatment with CDFs exhibit regular surface. However, after incubating with CDFs, the morphology of the bacterial cells including *S. aureus* (Fig. 8b) an*d E. coli* (Fig. 8d) changed drastically. Moreover, the membranes of many bacterial cells broke apart. Some small materials were observed on the surface of the bacteria, which originated from the attached CDFs. This indicates the CDFs can disinfect the bacterial cells by damaging the membranes [32].

**Fig. 9:**
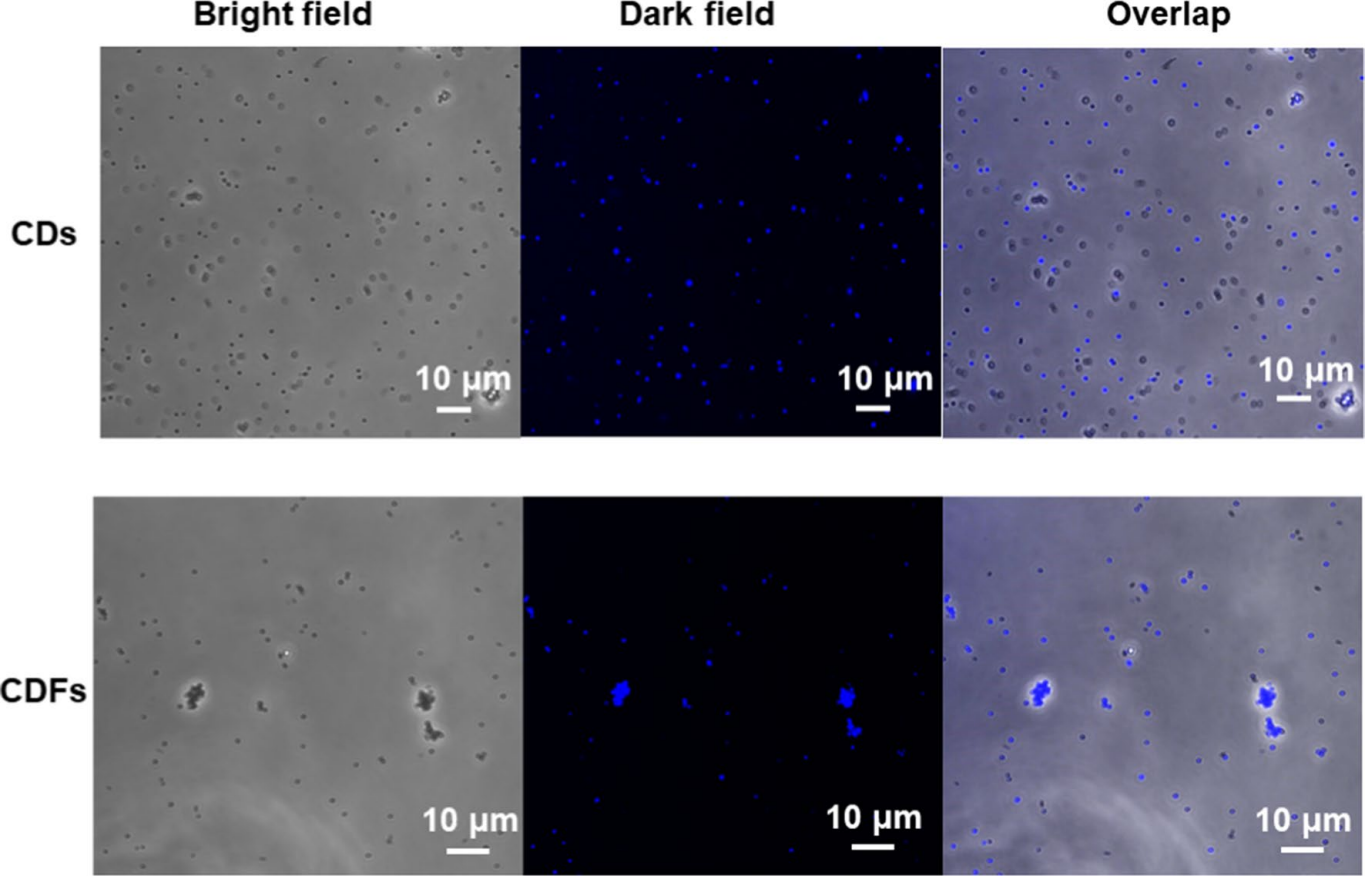
Imaging of *S. aureus* by the uptake of CDs and CDFs after 12 h

Since both CDs and CDFs are fluorescent, 6 µg/mL of the agents was investigated for bacterial imaging during the progress of disinfection. The imaging of *S. aureus* was investigated and shown in Fig. 9. Interestingly, it was found that both CDs and CDFs could be used for imaging of the *S. aureus*. However, CDFs show higher uptake efficiency since the bacterial cells observing from the bright and dark fields almost overlapped. On the other hand, only part of *S. aureus* cells was stained by the CDs. Besides, the bacterial cell densities were smaller by the treatment of CDFs, representing CDFs disinfected *S. aureus* more efficiently compared to CDs at the same dosages. These results also reveal that CDFs may be used as alternative dyes for imaging various bacterial cells.

## Conclusions

The assembly of CDs into CDFs results in more robust antibacterial activity. It is concluded that the assembly structure enables more stable properties but magnifies the antibacterial activity of CDs. This work also provides a new avenue of assembly small nanomaterials into frameworks for more practical applications.

## Supplementary Information

**Additional file 1. **The following are available online at www.mdpi.com/xxx/s1, **Figure S1:** TEM (a),HR-TEM (b), and AFM (c, d) of the as-obtained CDs; **Figure S2:** SEM of CDscharacterized at four random times; **Figure S3:** (a) UV-vis, (b) the fluorescenceexcitation and emission spectra of the CDs;** Figure S4:** XPS survey (a) and FTIR ofthe as-obtained CDs; **Table S1:** Lifetime of the CDs and CDFs.

## Data Availability

All data supporting the conclusions of this article are included within the article.

## Abbreviations

CDs: Carbon dots
CDFs: Carbon dots-based frameworks
PEI: Polyethylenimine
*E. coli*: *Escherichia coli*
*S. aureus*: *Staphylococcus aureus*
TEM: Transmission electron microscope
HR-TEM: High resolution TEM
XPS: X-ray surface photoelectron spectra
MIC: Minimum inhibitory concentration
NCs: Nanoclusters
TCSPC: Time-correlated single-photon counting
CCK-8: Cell counting Kit-8 assay
ROS: Ros reactive oxygen species
SEM: Scanning electron microscope
ZP: Zeta potential
