# Correction: Assembly of Carbon Dots into Frameworks with Enhanced Stability and Antibacterial Activity

**DOI:** 10.1186/s11671-022-03751-y

**Published:** 2022-12-16

**Authors:** Pengfei Zhuang, Kuo Li, Daoyong Li, Haixia Qiao, Yifeng E, Mingqun Wang, Jiachen Sun, Xifan Mei, Dan Li

**Affiliations:** 1 Jinzhou Medical University, Jinzhou, China; 2 Department of Basic Science, Jinzhou Medical University, Jinzhou, China; 3 Department of Pharmacology, Jinzhou Medical University, Jinzhou, China; 4 Department of Basic Medical Science, Jinzhou Medical University, Jinzhou, China

**Correction: Nanoscale Res Lett (2021) 16:121** 10.1186/s11671-021-03582-3

Following publication of the original article [1], the authors flagged an error in affiliation 1: ‘Department of Orthopedics, The First Affiliated Hospital of Jinzhou Medical University, Jinzhou, China’ had been detailed instead of ‘Jinzhou Medical University, Jinzhou, China’.

The affiliation has now been corrected in the published article. The authors thank you for reading and apologize for any inconvenience caused.
