# Supplementary material for: Assembly of Carbon Dots into Frameworks with Enhanced Stability and Antibacterial Activity
Source: Nanoscale Res Lett. 2021 Jul 29;16:121. doi: 10.1186/s11671-021-03582-3 (PMC8322205; doi:10.1186/s11671-021-03582-3)
Supplement: Supplementary file 1 — Additional file 1. The following are available online at www.mdpi.com/xxx/s1, Figure S1: TEM (a),HR-TEM (b), and AFM (c, d) of the as-obtained CDs; Figure S2: SEM of CDscharacterized at four random times; Figure S3: (a) UV-vis, (b) the fluorescenceexcitation and emission spectra of the CDs; Figure S4: XPS survey (a) and FTIR ofthe as-obtained CDs; Table S1: Lifetime of the CDs and CDFs. [file 11671_2021_3582_MOESM1_ESM.docx]

Assembly of carbon dots into frameworks with enhanced stability and antibacterial antibacterial activity

Pengfei Zhuang^1, 2^, Kuo Li^1^, Daoyong Li^1^, Haixia Qiao^2^, Yifeng E^3^, Mingqun Wang^4^, Jiachen Sun^1^, Xifan Mei^1^* and Dan Li^1, 2^*

^1^ Department of Orthopedics, the First Affiliated Hospital of Jinzhou Medical University, Jinzhou, China;

^2^ Department of Basic Science, Jinzhou Medical University, Jinzhou, China;

^3^ Department of pharmacology, Jinzhou Medical University, Jinzhou, China；

^4^ Department of Basic Medical Science, Jinzhou Medical University, Jinzhou, China；

*** Correspondence:**

meixifan@jzmu.edu.cn; danli@jzmu.edu.cn

The morphology and size of the as-obtained carbon dots (CDs) were characterized by transmission electron microscopy (TEM) and atomic force microscope (AFM) as shown in Figure S1. The CDs are initially nearly spherical with an average diameter smaller than 5.4 nm. However, after a long time of exposure to the air, the mono-dispersed morphology could not be kept.


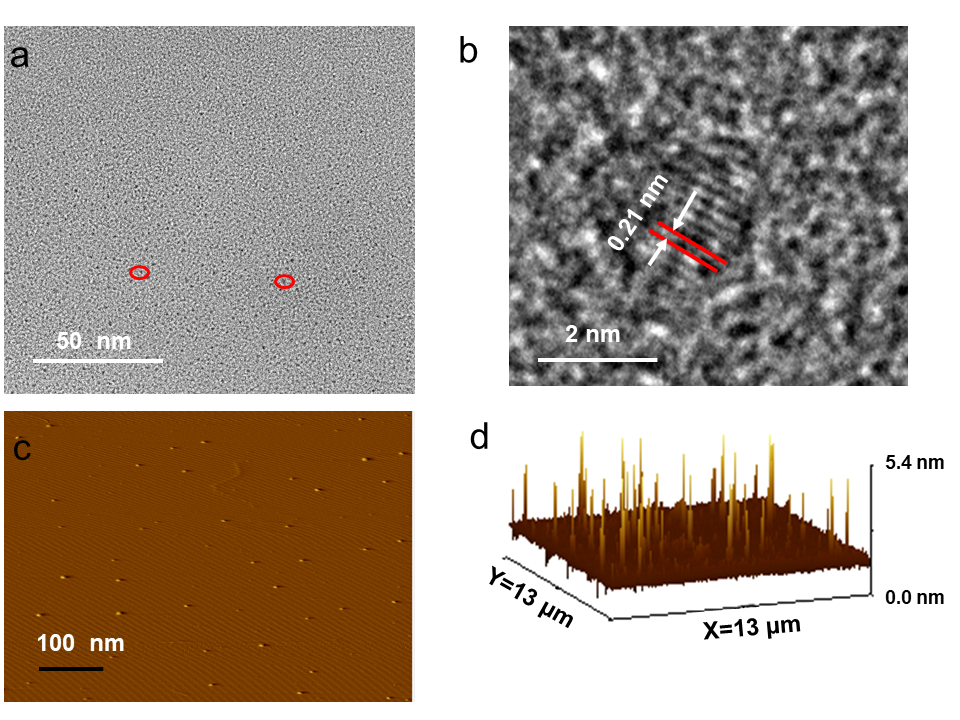


**Figure S1.** TEM (a), HR-TEM (b) and AFM (c, d) of the as-obtained CDs.


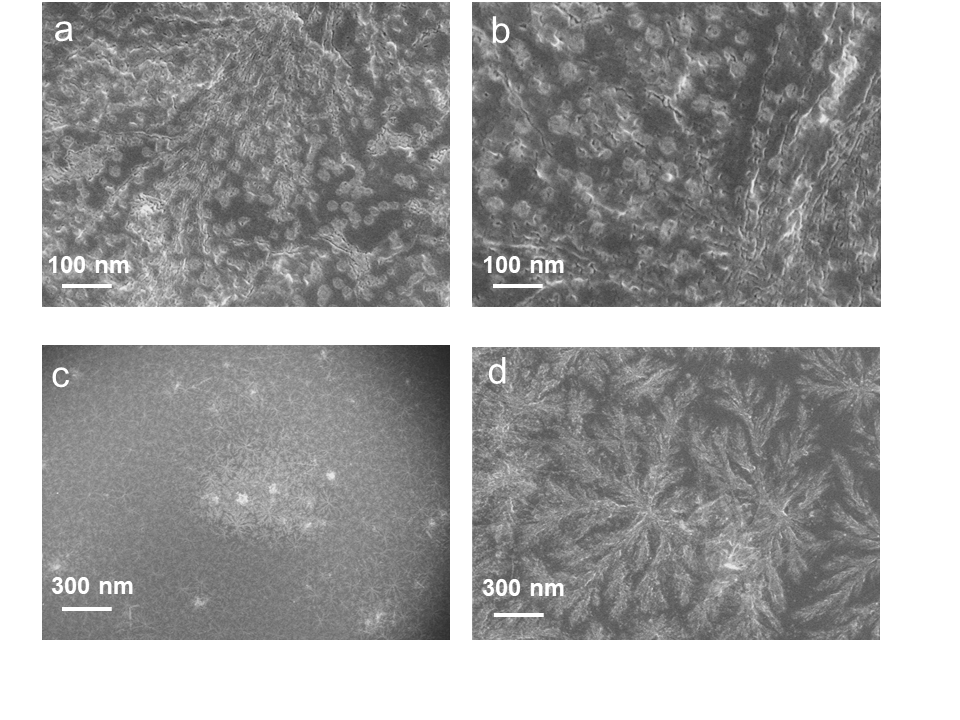


**Figure S2.** SEM of CDs characterized at four random times.

The optical properties of the CDs were investigated using the UV-visible (UV-vis) absorption and fluorescence spectra at room temperature (Figure S2). No obvious absorption above 240 nm for L-cysteine was obtained for the UV-vis spectra (Figure S2a), indicating the formation of CDs. The fluorescence spectra of the CDs showed the maximum emission at approximately 430 nm with 350 nm excitation (Figure S2b).


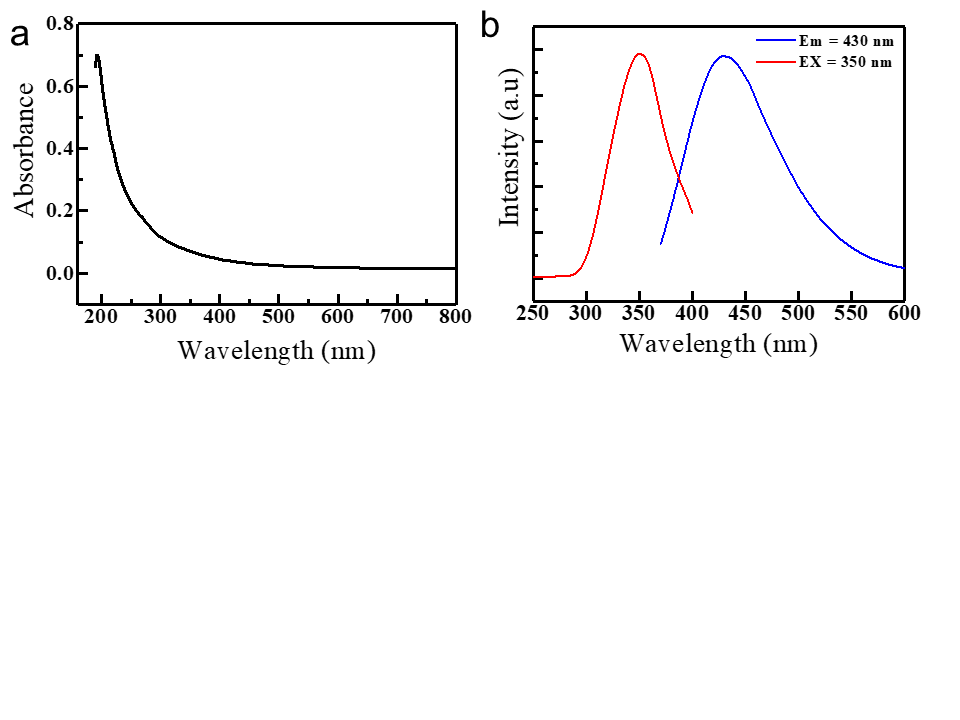


**Figure S3.** (a) UV-vis, (b) the fluorescence excitation and emission spectra of the CDs.

The X-ray surface photoelectron spectra (XPS) results indicate that the CDs are mainly composed of carbon (C), nitrogen (N), sulfur (S), and oxygen (O) (Figure 1A). Fourier transform infrared spectroscopy (FTIR) analysis of CDs showed the stretching vibration signals of C-N, N-H, S-H, C-O-C, C-S, C-N. These results indicated that various carboxyl and amine functional groups were present on the surfaces of CDs.


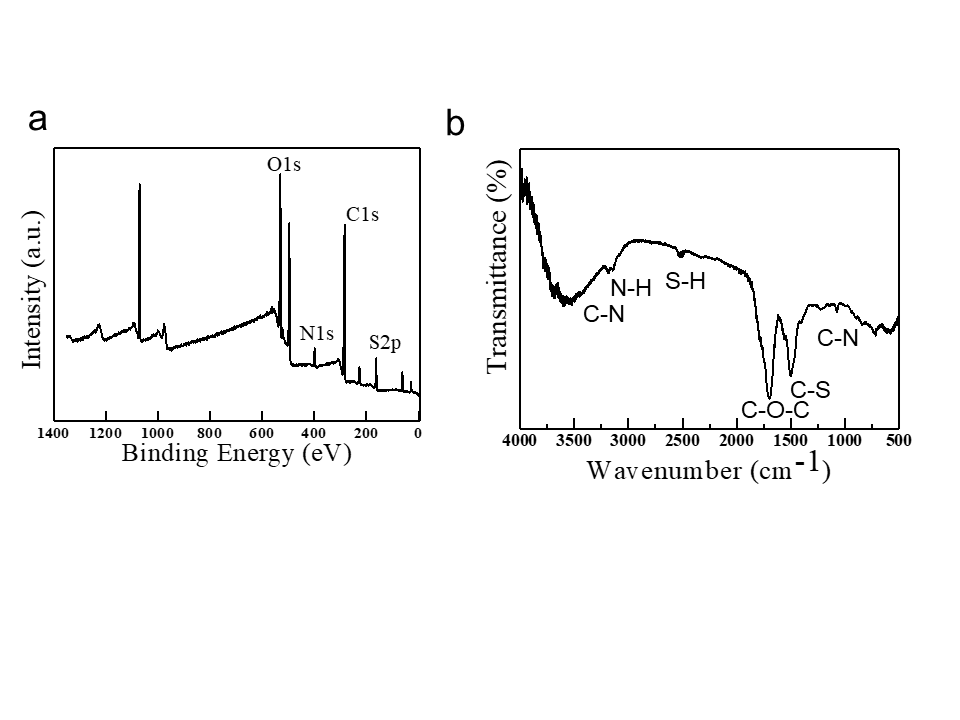


**Figure S4.** XPS survey (a) and FTIR of the as-obtained CDs.

The fluorescence decay curves are fitted with a sum of two exponential functions (Table S1).

I=A+B1exp(-t/t_1_)+B2exp(-t/t_2_)

**Table S1** Lifetime simulation parametrial of CDs and CDs-based frameworks (CDFs)

|  | B1 | t1 (ns) | B2 | t2 |
| --- | --- | --- | --- | --- |
| CDs | 1134.16 | 2.45 | 428.46 | 7.47 |
| CNS | 1128.67 | 1.98 | 373.4709 | 7.30 |
